# Prozessmodularisierung und -standardisierung als Grundlage für die Digitalisierung von Prozessen im Öffentlichen Gesundheitsdienst

**DOI:** 10.1365/s40702-022-00914-4

**Published:** 2022-10-27

**Authors:** Artemiy von Tsurikov, Martin Engert, Andreas Hein, Helmut Krcmar

**Affiliations:** Technische Universität München, Boltzmannstraße 3, 85748 Garching, Deutschland

**Keywords:** Öffentlicher Gesundheitsdienst, Öffentliche Verwaltung, Öffentlicher Sektor, Digitalisierung, E‑Government, Prozessstandardisierung, Prozessmodularisierung, Public health service, Public administration, Public sector, Digitization, E‑Government, Process standardization, Process modularization

## Abstract

Die Digitalisierung des Öffentlichen Gesundheitsdienstes (ÖGD) in Deutschland weist nach wie vor Defizite auf, was nicht zuletzt im Kontext der Pandemiebewältigung zu Problemen führte. Zur Bewältigung ihrer Aufgaben benötigen die etwa 400 deutschen Gesundheitsämtern, eine entsprechende Softwareausstattung, deren Bereitstellung auch durch die Heterogenität des ÖGD erschwert wird. Entsprechend stellt die Standardisierung und Modularisierung von Prozessen die Grundlage für eine nachhaltige Digitalisierung des ÖGD dar. Aufbauend auf diesen Prozessmodellen können geeignete und breit anwendbare Softwaremodule für diverse Aufgabenbereiche der Gesundheitsämter entwickelt und bereitgestellt werden. Die durchgeführte Studie leistet einen ersten wichtigen Beitrag im Kontext der Digitalisierung des ÖGD, indem sie eine Vorgehensweise zur Prozessstandardisierung und -modularisierung entwickelt und beispielhaft anwendet. Im Rahmen dieser Studie erfolgte eine Standardisierung und Modularisierung von Prozessen im Bereich der Wasserhygiene basierend auf den Prozessen eines Gesundheitsamtes in Berlin. In einer Kombination von Design Science Research und Business Process Standardization (BPS) wurden stakeholderübergreifende Ende-zu-Ende Prozesse identifiziert, deren Ist-Zustände mit BPMN (Business Process Model and Notation) visualisiert und anschließend in generalisierbare Soll-Zustände überführt. Die Ergebnisse wurden schließlich mit zwei weiteren Gesundheitsämtern validiert. Die angewandte Methodik und die entwickelten Artefakte können einerseits zur Skalierung dieses Vorgehens in weiteren Gesundheitsämtern und andererseits als eine Grundlage für die Entwicklung von passenden und breit anwendbaren Softwarekomponenten genutzt werden.

## Abstract (en)

The digitization of the Public Health Service in Germany still has deficits, which led to problems, not least in the context of pandemic management. To address their responsibilities, the approximately 400 German local health authorities require appropriate software equipment, which is exacerbated by the heterogeneity of the Public Health Service. Consequently, the standardization and modularization of processes represent the basis for sustainably digitizing the Public Health Service. Building on the associated process models, suitable and broadly applicable software modules can be developed and made available for diverse areas of responsibility of the public health departments. In this study, we standardize and modularize water hygiene processes of one local health authority in Berlin, Germany. Combining Design Science Research and Business Process Standardization (BPS), we identified cross-stakeholder end-to-end processes, visualized their actual states with BPMN (Business Process Model and Notation), and transferred them into generalizable target states. We finally validated the results with two other local health authorities in Germany. The applied methodology and the developed artifacts can be used to scale this approach in further health departments and build the basis for developing suitable and widely applicable software components.

## Einleitung

Die Corona-Krise hat die Digitalisierungsdefizite in ganz Deutschland (Scheiber et al. 2020) und insbesondere im öffentlichen Gesundheitsdienst (ÖGD) offengelegt (Parlamentarisches Begleitgremium COVID-19-Pandemie 2021). Bedingt durch die Komplexität föderaler Strukturen und die Unterfinanzierung des ÖGD konnten die für die Pandemie dringend benötigten digitalen Werkzeuge und Standards, mit denen Interoperabilität und Datenaustausch möglich gewesen wären, nicht entwickelt werden. Während einige Gesundheitsämter sich eigenständig durch improvisierte Lösungen und Workarounds helfen konnten, mussten fehlende digitale Prozesse durch den zusätzlichen Einsatz von Personal kompensiert werden (Zimmermann et al. 2021).

Der ÖGD bildet neben der ambulanten und der stationären medizinischen Versorgung eine der drei Säulen des deutschen Gesundheitswesens. Der ÖGD unterteilt sich in Deutschland in die Bundesebene, die Länderebene und die Kommunalebene. Jede dieser Ebenen wird durch unterschiedliche Behörden repräsentiert. Auf Bundesebene übernimmt beispielsweise das Robert-Koch-Institut zentrale Aufgaben der öffentlichen Gesundheitspflege im Bereich Infektionskrankheiten. Auf Länderebene werden diese Aufgaben von Landesämtern oder Gesundheitsministerien wahrgenommen, während auf kommunaler Ebene rund 400 Gesundheitsämter als bevölkerungsnahe Behörden sich um Beratung, Schutz und Prävention in der Bevölkerungsmedizin kümmern (Akademie für Öffentliches Gesundheitswesen Akademie für Öffentliches Gesundheitswesen Düsseldorf 2019).

Einzelne Gesundheitsämter unterscheiden sich in ihren Funktions- und Arbeitsweisen. Diese Unterschiede entstehen häufig durch regionale und bundeslandabhängige Spezifitäten, die sich in Form von strukturellen und gesetzlichen Rahmenbedingungen zeigen (Deutscher Bundestag 2021). Zudem spielen Unterschiede in der technischen und der personellen Ausstattung der Gesundheitsämter eine wichtige Rolle. Solche Unterschiede führen dazu, dass die Zusammenarbeit und die Koordination der Gesundheitsämter deutlich erschwert wird. Daraus entsteht ein Bedarf zur Entwicklung einheitlicher und digitaler Lösungen im Rahmen der Digitalisierung des ÖGD (Scholta et al. 2019). Zusätzlich hat die Homogenisierung und Digitalisierung vor dem Hintergrund des *Onlinezugangsgesetzes (OZG)*, welches die Digitalisierung aller Verwaltungsdienstleistungen mit Schnittstelle zu Anwendern vorsieht, an Bedeutung gewonnen (Bundesministerium des Innern und für Heimat 2022; Halsbenning 2021).

Zur Lösung dieser Herausforderung hat der Bund im vergangenen Jahr den *Pakt für den Öffentlichen Gesundheitsdienst* in Höhe von 4 Mrd. € verabschiedet. Davon steht ein Budget von 800 Mio. € für die Digitalisierung des ÖGD zur Verfügung. Diese Mittel sollen Maßnahmen zur Digitalisierung des ÖGD sowohl auf Landesebene als auch auf Kommunalebene fördern (Bundesministerium für Gesundheit 2021). Das Konzept des Bundesministeriums für Gesundheit sieht vor, dass jede Einrichtung des ÖGD zunächst den aktuellen Digitalisierungsstand selbstständig bestimmt und diesen anschließend gezielt durch die Digitalisierung von Prozessen vorantreibt. Hierbei bekommen die Kommunen Unterstützung seitens des Bundesministeriums und haben zudem die Möglichkeit, in Zusammenarbeit mit anderen Kommunen Digitalisierungsprojekte anzugehen und finanzielle Mittel zur Unterstützung ihrer Vorhaben aus dem festgelegten Budget zu beantragen. Bei diesen Digitalisierungsprojekten sollte der Fokus primär auf der Entwicklung standardisierter Softwaremodule zur Unterstützung der Prozesse in den Gesundheitsämtern liegen.

Offen ist jedoch die Frage, wie die zu entwickelnden Softwaremodule basierend auf gesetzlichen Vorgaben (bspw. Infektionsschutzgesetz) standardisiert entwickelt werden können, um eine möglichst hohe Nachnutzung in den rund 400 Gesundheitsämtern zu ermöglichen. Die Erfahrungen mit der Einführung des „Surveillance Outbreak Response Management and Analysis System“ (SORMAS) zur epidemiologischen Kontrolle und Nachverfolgung des COVID-19 Virus hat gezeigt, dass die Heterogenität der Systemlandschaften und die dadurch jeweils unterschiedlichen Prozesse die Gesundheitsämter vor massive Probleme bei der Einführung stellen (Deutscher Bundestag 2021; Zimmermann et al. 2021). Für die Realisierung homogener und wiederverwendbarer Softwaremodule ist daher eine Standardisierung und Modularisierung der grundlegenden Prozesse in den Gesundheitsämtern notwendig. Die durchgeführte Studie liefert hierfür den ersten wichtigen Beitrag, indem sie ein Vorgehen zur Prozessstandardisierung und -modularisierung am Beispiel eines Berliner Gesundheitsamtes im Bereich der Wasserhygiene bietet.

## Methodik und Vorgehen

**Abb. 1:**
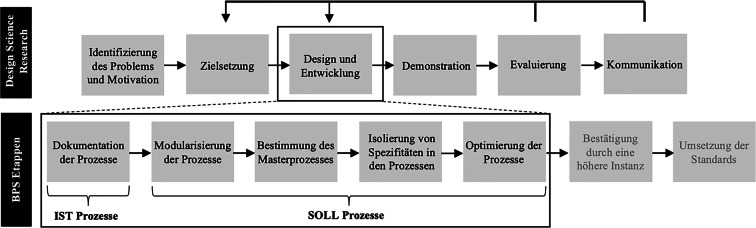
Konzeptionelle Darstellung der angewandten Methodik Quelle: Eigene Darstellung in Anlehnung an Peffers et al. (2007) und Goel et al. (2021)

Die methodische Grundlage für die Standardisierung und Modularisierung der Prozesse bildete der *Design Science Research* Prozess nach Peffers et al. (2007). Das Design Science Paradigma beruht darauf, Forschung durch das Erstellen von neuen und innovativen Artefakten zu betreiben (Hevner et al. 2004). Dieser Ansatz wurde zusätzlich mit einem *Business Process Standardization (BPS) *Ansatz nach Goel et al. (2021) kombiniert (Abb. 1). Goel et al. (2021) erstellen eine literaturbasierte Typologie von zwölf BPS-Strategien und beschreiben sieben durchzuführende Etappen. Design Science Research bildet somit den übergeordneten Rahmen dieser Studie, während die BPS-Etappen die inhaltlichen Arbeitsschritte vorgeben.

Die durchgeführte Studie wurde durch das vorherrschende Problem der Heterogenität der Systemlandschaften und Prozesse in den Gesundheitsämtern und zusätzlich durch den Vorschlag zur Lösung dieses Problems durch die Entwicklung eines virtuellen Gesundheitsamtes angestoßen. Um sich einer Lösung für diese Problemstellung anzunähern, wurde als Ziel die beispielhafte Durchführung einer Standardisierung und Modularisierung von Prozessen im Bereich der Wasserhygiene eines Berliner Gesundheitsamtes festgelegt. Anhand des Beispiels sollte eine generische Vorgehensweise entwickelt werden, welche sich auf andere Gesundheitsämter und Prozesse übertragen ließe.

Beim Design und der Entwicklung der Artefakte wurden zunächst die vorhandenen Prozesse im Bereich der Wasserhygiene des Berliner Gesundheitsamtes mithilfe von BPMN (Business Process Model and Notation) dokumentiert. Die modellierten Ist-Prozesse beruhen auf Daten von insgesamt neun Interviews mit den wichtigsten Interessensgruppen und bilden alle am Prozess beteiligten Stakeholder, die relevanten Abläufe sowie die im Prozess verarbeiteten Daten und die dafür verwendeten IT-Systeme ab. Nach der Fertigstellung der Ist-Prozesse erfolgte die Demonstration und Evaluierung der Prozesse mit den Experten des Gesundheitsamtes. Hierbei wurden die modellierten Prozesse entlang ihres Ablaufs durchgesprochen und Verbesserungen sowie Ergänzungen vorgenommen. Die Evaluierungsrunde führte iterativ zurück zum Design und Entwicklung und somit zur Anpassung der Modelle. Eine zusätzliche Analyse und Identifikation von Schwachstellen und Optimierungspotenzialen bildete die Grundlage für die weitere Erarbeitung der optimierten Soll-Prozesse.

Für die Erarbeitung der Soll-Prozesse wurden die modellierten Prozesse durch Modularisierung in kleinere Subprozesse aufgespalten. Unter den resultierenden Prozessmodulen gab es welche, die einmalig vorkamen, und andere, die in mehreren Prozessen vorhanden waren und unterschiedliche Ausprägungen aufwiesen. Um die Standardisierung effektiv durchführen zu können, wurde für jedes Prozessmodul ein Masterprozess als ein Referenzpunkt bestimmt. Im weiteren Vorgehen wurden in den Masterprozessen Spezifitäten und Besonderheiten abstrahiert, um alle standardisierten Prozesse zu einem gewissen Grad anpassbar und generalisierbar zu machen. Letztlich wurden die identifizierten Prozessmodule in Anlehnung an die Masterprozesse optimiert. Hierbei wurden die in den Ist-Zuständen identifizierten Schwachstellen und Optimierungspotenziale mithilfe einer geeigneten Lösung behoben. Die Ergebnisse der modularisierten, standardisierten und optimierten Prozessmodule wurden ebenfalls mithilfe von BPMN modelliert, dem Gesundheitsamt vorgestellt, mit Gesundheitsaufsehern evaluiert und angepasst. Zusätzlich wurden zwei Evaluierungsrunden mit weiteren Gesundheitsämtern in Deutschland durchgeführt, um die Übertragbarkeit und die Generalisierbarkeit der erarbeiteten Soll-Prozesse zu bekräftigen.

Da sich die Standardisierung und Modularisierung der Prozesse nur auf das Gesundheitsamt in Berlin beschränkte und im Rahmen dieser Arbeit nur eine mögliche Lösung erarbeitet, jedoch nicht umgesetzt wurde, sind die beiden letzten Schritte entfallen. Durch das Festhalten des Problems, der Zielsetzung, des Vorgehens und der Ergebnisse in dieser Studie wurden letztlich die Ergebnisse an die beteiligten Interessensgruppen kommuniziert.

## Ergebnisse der Prozessmodularisierung und -standardisierung

Gesundheitsämter in Deutschland verfügen über ein breites Aufgabenspektrum, welches unter anderem von biologischen Arbeitsstoffen, Gesundheitsberichterstattung und Gesundheitshilfen für Kinder und Jugendliche, dem Infektionsschutz und sozialposychatrischen Dienst bis zur Umweldmedizin reicht. Für den Kontext dieser Studie wurde das Aufgabenfeld Wasserhygiene ausgewählt.

Im Laufe der Studie wurden im Bereich der Wasserhygiene des Berliner Gesundheitsamtes insgesamt 14 Prozesse identifiziert und sieben Themenbereichen zugeordnet (Tab. 1). Jeder Themenbereich beruht auf einer gesetzlichen Grundlage, wie etwa der Trinkwasserverordnung (TrinkwV), dem Wasserhaushaltsgesetz (WHG), dem Wassersicherstellungsgesetz (WasSiG) und dem Infektionsschutzgesetz (IfsG).

**Tab. 1:** Übersicht über die Themenbereiche und identifizierten Prozesse

Nr	Themenbereich	ID	Identifizierte Prozesse
1	Rohrnetzstellen nach § 3 Abs. 2a TrinkwV (A-Anlagen)	P1	Untersuchung von Rohrnetzstellen
2	Dezentrale kleine Wasserwerke nach § 3 Abs. 2b TrinkwV (B-Anlagen)	P2	Untersuchung von kleinen dezentralen Wasserwerken
		P3	Änderung des Untersuchungsrhythmus und Parameter bei der Untersuchung von kleinen dezentralen Wasserwerken
		P4	Übermittlung der Befunde an das Landesgesundheitsamt im Rahmen der EU-Berichterstattung
3	Kleinanlagen zur Eigenversorgung nach § 3 Abs. 2c TrinkwV (C-Anlagen)	P5	Untersuchung von Kleinanlagen zur Eigenversorgung
		P6	Abbau von Zapfstellen bei Kleinanlagen zur Eigenversorgung
4	Mobile Versorgungsanlagen nach § 3 Abs. 2d TrinkwV (D-Anlagen)	P7	Untersuchung von mobilen Versorgungsanlagen^a^
5	Hausinstallationen nach § 3 Abs. 2e TrinkwV (E-Anlagen)	P8	Untersuchung von Hausinstallationen auf Legionellen
		P9	Anlassbezogene Untersuchung von Hausinstallationen auf Legionellen im Falle eines Legionellosen Krankheitsfalles
		P10	Erhöhung des Untersuchungsrhythmus bei der Untersuchung von Hausinstallationen auf Legionellen
6	Straßenbrunnen zur Notwasserversorgung Nach § 50 Abs. 1 WHG und § 1 Abs. 1 Nr. 1 WasSiG	P11	Untersuchung von Straßenbrunnen
7	Frei- und Hallenbäder nach § 37 Abs. 2 IfsG	P12	Untersuchung von Frei- und Hallenbädern initiiert durch den Betreiber
		P13	Untersuchung von Frei- und Hallenbädern initiiert durch das Gesundheitsamt
–	Themenübergreifend	P14	Fristen verfolgen (relevant für Themenbereich Nr. 2, 3, 4, 5, 7)

^a^Identisch zur Untersuchung von Kleinanlagen zur Eigenversorgung (C-Anlagen)

Quelle: Eigene Darstellung

Zudem lassen sich die identifizierten Prozesse in drei Gruppen gliedern. *Untersuchungsprozesse *(P1, P2, P5, P7, P8, P9, P11, P12, P13) kommen in jedem Bereich vor und bilden die Hauptprozesse. Man versteht unter den Untersuchungsprozessen die Untersuchung des Wassers in Wasserversorgungsanlagen auf bestimmte mikrobiologische, chemische oder physikalische Parameter. *Hintergrundprozesse *(P3, P6, P10, P14) sind ergänzende Prozesse zu den Untersuchungsprozessen, bei denen bestimmte Abläufe passieren, die Auswirkungen auf die Untersuchungsprozesse haben können. Dies kann zum Beispiel die Änderung von Untersuchungsrhythmen oder Untersuchungsparametern sein. Die letzte Gruppe bilden die *Meldeprozesse *(P4 und als Subprozess in P9, P11). Meldeprozesse sind Prozesse, bei denen bestimmte Informationen vom Gesundheitsamt an weitere öffentliche Institutionen wie zum Beispiel das Landesamt übermittelt werden.

Mithilfe der verwendeten BPS-Methodik konnten im Verlauf der Studie in den 14 identifizierten Prozessen insgesamt 10 Prozessmodule herausgearbeitet werden (Tab. 2). Module sind Subprozesse der ursprünglichen Prozesse, die eine optimierte und standardisierte Variante aller vorkommenden Variationen eines Moduls darstellen. Dank der Ausrichtung der Module an Referenzprozessen liegen diese in einer standardisierten Ausprägung vor und sind aus diesem Grund homogen nach innen. Untereinander sind die einzelnen Module jedoch heterogen, da sie diverse Subprozesse des ursprünglichen Prozesses visualisieren. Aufgrund dieser Art der Gestaltung der Module ist es möglich, aus den einzelnen Modulen rückläufig die ursprünglichen Prozesse in einer standardisierten und optimierten Form zusammenzusetzen. Zudem erlauben die Module durch deren Kombination die Zusammensetzung neuer Prozesse, die auf standardisierten Bausteinen fundieren.

**Tab. 2:** Identifizierte Prozessmodule

ID	Modul	Prozesse, in denen das Modul vorkommt
PM1	Initiierung und Durchführung einer Untersuchung mit anschließender Meldung an das Gesundheitsamt	P1, P2, P5, P7, P8, P9, P11, P12
PM2	Initiierung und Durchführung einer amtlichen Untersuchung mit anschließender Meldung an das Gesundheitsamt	P13
PM3	Erfassung von einem Labor übermittelter Daten im Gesundheitsamt	P1, P2, P5, P7, P8, P9, P11, P12, P13
PM4	Meldung und Ermittlung eines Legionellosen Krankheitsfalles im Gesundheitsamt	P9
PM5	Kontaktaufnahme des Gesundheitsamtes zum Betreiber	P2, P3, P5, P6, P7, P8, P9, P10, P11, P12, P13, P14
PM6	Übermittlung von Befunden an weitere Ämter durch das Gesundheitsamt	P4, P11
PM7	Bearbeitung eines Antrags auf Erhöhung des Untersuchungsrhythmus	P10
PM8	Meldung des entnommenen Wasservolumens durch einen Betreiber an das Gesundheitsamt	P3
PM9	Begutachtung von abgebauten Zapfstellen bei einer Kleinanlage zur Eigenwasserversorgung	P6
PM10	Verfolgen von Fristen im Gesundheitsamt	P14

Quelle: Eigene Darstellung

**Abb. 2:**
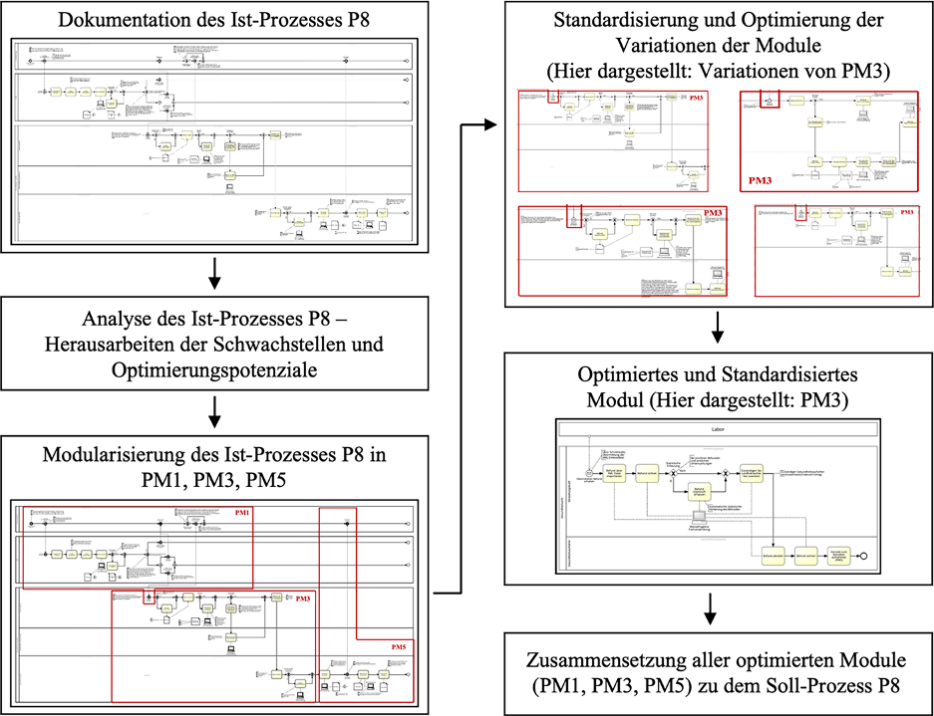
Transformation eines Ist-Prozesses in einen Soll-Prozess. Quelle: Eigene Darstellung

Um das Vorgehen und die Ergebnisse zu illustrieren, wird im Folgenden die Transformation eines Ist-Prozesses in einen Soll-Prozess anhand von einem der 14 identifizierten Prozesse gezeigt (Abb. 2). Bei dem ausgewählten Prozess handelt es sich um den Untersuchungsprozess von Hausinstallationen auf Legionellen (P8). Dieser Prozess lässt sich dem Themenbereich der Untersuchung von Hausinstallationen (E-Anlagen), die in § 3 Abs. 2e TrinkwV gesetzlich festgelegt sind, zuordnen.

### Dokumentation des Ist-Prozesses

Die wesentlichen Akteure in diesem Prozess sind die Hausverwaltung als der Betreiber, das Labor, welches die Wasseruntersuchung durchführt und das Gesundheitsamt. Der ausgewählte Prozess wird durch eine Untersuchungspflicht initiiert. Dabei wird zunächst vom Betreiber an ein Labor der Auftrag für eine Untersuchung vergeben. Das Labor stellt den Probenehmer, der die Probe entnimmt. Anschließend wird die Probe im Labor untersucht und der Untersuchungsbefund erstellt. In Abhängigkeit von dem Ergebnis des Befundes wird der Befund gegebenenfalls an das Gesundheitsamt übermittelt. Im Falle einer Übermittlung des Befundes an das Gesundheitsamt wird der Befund in einer Reihe von Schritten von Verwaltungskräften und Gesundheitsaufsehern erfasst, begutachtet und führt gegebenenfalls zur Kontaktaufnahme mit dem Betreiber und der Anordnung von weiteren Maßnahmen.

### Analyse des Ist-Prozesses

Im Rahmen der Analyse des Ist-Prozesses war es auf Grundlage von Erkenntnissen über die beteiligten Akteure, Abläufe, Kommunikationswege, Kommunikationsmittel und Arten der genutzten Daten sowie IT-Systeme zur Datenverarbeitung möglich, wesentliche Schwachstellen und Optimierungspotenziale in diesem Prozess herauszuarbeiten. Zu solchen Schwachstellen gehörten unter anderem inkonsistente Datenformate bei der Übermittlung von Befunden vom Labor an das Gesundheitsamt, erforderliche manuelle Eingaben im Gesundheitsamt bei der Erfassung von Befunden, manuelles Erstellen von Schreiben durch die Gesundheitsaufseher, diverse und inkonsistente Kommunikationsmittel zwischen den Akteuren sowie unterschiedliche Arbeitsweisen des Personals im Gesundheitsamt, die zu Redundanz und Inkonsistenz in Daten und Abläufen führten. Diese Erkenntnisse bildeten die Grundlage für die anschließende Optimierung der Prozesse.

### Modularisierung des Ist-Prozesses

Die Modularisierung des Prozesses erlaubte es, die Komplexität zu reduzieren und einen bestimmten Subprozess hervorzuheben. Die Bestimmung der gekennzeichneten Module richtet sich sowohl nach wiederkehrenden und ähnelnden Prozessabschnitten zwischen den unterschiedlichen Prozessen als auch nach logischen Prozessabschnitten innerhalb des betrachteten Prozesses. Der gewählte Untersuchungsprozess auf Legionellen lässt sich konkret in drei Module aufteilen. Das erste Modul beschreibt den Subprozess, bei dem der Betreiber einen Untersuchungsauftrag an das Labor vergibt, welches den Untersuchungsauftrag ausführt und anschließend den Befund an den Betreiber und gegebenenfalls das Gesundheitsamt übermittelt (PM1). Das zweite Modul beinhaltet das Empfangen eines Befundes im Gesundheitsamt und die Erfassung dieses Befundes im System sowie dessen Begutachtung (PM3). Das letzte Modul beinhaltet die Kommunikationsaufnahme des Gesundheitsamtes zum Betreiber und die mögliche Anordnung weiterer Maßnahmen (PM5).

### Standardisierung und Optimierung der Variationen der Module

Die herausgearbeiteten Module kamen mehrfach in den identifizierten Prozessen in unterschiedlichen Ausprägungen vor (Tab. 2). Für die Homogenisierung und Standardisierung der Prozesse war es notwendig, alle Ausprägungen der Module einem optimierten Referenzprozess anzugleichen. Der optimale Zustand eines Prozesses wurde insbesondere durch die zuvor identifizierten Optimierungspotenziale bestimmt. Somit konnte zum Beispiel durch die Verwendung von standardisierten Datenformaten und einem automatischen Import über eine Schnittstelle die Übermittlung und Erfassung der Befunde im Gesundheitsamt optimiert werden. Des Weiteren konnte durch die Automatisierung von manchen Abläufen die Anzahl der manuellen Eingaben und manuellen Schritte reduziert werden, was die Fehlerquote senkte. Mithilfe von einheitlichen Schnittstellen war es zudem möglich, veraltete Kommunikationsmittel wie Fax und Post zu ersetzen und für eine einfachere, schnellere und standardisierte Kommunikation zu sorgen. Aus diesem Vorgehen resultierte somit ein standardisiertes und optimiertes Modul in seinem optimalen Soll-Zustand. Dieses konnte im Weiteren dafür verwendet werden, mit den restlichen beiden optimierten Modulen rückläufig den ganzen Soll-Prozess zusammenzusetzen und zusätzlich in Kombination mit optimierten Modulen aus anderen Prozessen neue Prozesse auf einer standardisierten Basis zu generieren.

Das aufgezeigte Vorgehen und die daraus resultierenden Ergebnisse anhand des ausgewählten Prozesses sind nur ein Ausschnitt aus dem, was im Rahmen der Studie durchgeführt wurde. Ein analoges Vorgehen wurde in den restlichen 13 Prozesse eingesetzt, um Module ausfindig zu machen, diese zu optimieren, zu standardisieren und daraus die Soll-Prozesse abzuleiten. Darüber hinaus wurde in Zusammenarbeit mit zwei weiteren Gesundheitsämtern die Übertragbarkeit der Methodik und die Generalisierbarkeit der ausgearbeiteten Module ausgewertet. Es stellte sich dabei heraus, dass die angewandte Methodik ein nützliches Instrument für potenzielle Anwender ist. Zudem konnten in den Modulen viele Gemeinsamkeiten auf prozessualen Ebenen sowie Gemeinsamkeiten in Bezug auf die vorherrschenden Schwachstellen und daraus resultierenden Optimierungspotenziale gefunden werden. Daraus lässt sich folgern, dass die erarbeiteten Artefakte handhabbar sind und im Weiteren als ein Referenzpunkt bei der Standardisierung und Modularisierung von Prozessen von weiteren Gesundheitsämtern genutzt werden können.

## Limitationen der Studie

Die Ausarbeitung der Prozessmodule erfolgte auf Grundlage der Prozesse im Bereich der Wasserhygiene, welcher jedoch nur ein Teil des breiten Aufgabenspektrums der Gesundheitsämter in Deutschland abbildet. Dies hat zu Folge, dass die erarbeitete Sammlung der Prozessmodule nicht vollständig ist und im Weiteren durch die Analyse weiterer Bereiche ergänzt werden kann. Darüber hinaus bilden die Prozesse des Berliner Gesundheitsamtes die Grundlage für diese Module. Eine analoge Analyse im gleichen Bereich anderer Gesundheitsämtern würde aufgrund der regionalen Unterschiede und der gesetzgebenden Spezifika voraussichtlich zu leicht abgewandelten und vor allem ergänzenden Modulen führen. Dem zu Folge ist es zurzeit schwer, die erarbeitete Sammlung als vollständigen zu bezeichnen. Des Weiteren stellte die Evaluierung der Ergebnisse mit zwei weiteren Gesundheitsämtern nur einen exemplarischen Ausschnitt eines Austausches und der Evaluierung zwischen den Gesundheitsämtern dar. Für die Beachtung aller region- und bundesspezifischen Gegebenheiten und eine signifikante Schlussfolgerung über die Übertragbarkeit der Ergebnisse braucht es eine größere und vor allem diverse Gruppe an Gesundheitsämter. Hinzu kommt, dass wie bereits in der Methodik geschildert, aufgrund des Umfangs der Studie nicht alle BPS-Etappen ausgeführt wurden. Dies hat zur Folge, dass die erarbeiteten und evaluierten Ergebnisse in der Theorie existieren, deren Umsetzbarkeit in der Praxis jedoch noch nicht bestätigt werden konnte.

## Verwendung der Ergebnisse und Skalierung des Vorgehens

Die durchgeführte Prozessmodularisierung und -standardisierung ist der erste Schritt auf dem Weg zur Entwicklung standardisierter und wiederverwendbarer Softwaremodule zur Unterstützung der Prozesse in den Gesundheitsämtern. Einheitliche und in wiederverwendbare Softwarekomponenten sind von großer Bedeutung, wenn es darum geht, die Gesundheitsämter und den ÖGD in Deutschland mit standardisierten Schnittstellen und mit einheitlicher, behördenübergreifender Infrastruktur auszustatten und somit für Interoperabilität zu sorgen (Behne und Teuteberg 2022). Basierend auf dieser Infrastruktur können grundsätzlich standardisierte Softwaremodule entwickelt und auf die jeweiligen Anforderungen jedes Gesundheitsamtes angepasst werden. Zusätzlich können bereits entwickelte Softwaremodule unter den Gesundheitsämtern nachgenutzt und geteilt werden, was den Ressourcenaufwand verringert und zur technischen sowie organisatorischen Standardisierung beiträgt. Insgesamt können dadurch Gesundheitsämter in Deutschland in die Lage versetzt werden, nach dem Baukastenprinzip die benötigten Softwaremodule entsprechend ihren Bedürfnissen und Zuständigkeiten zu nutzen und zu teilen. Die Lösung zeigt somit parallelen zu digitalen Plattformen, welche eine modular erweiterbare Infrastruktur darstellt, die von autonomen Akteuren (Hein et al. 2021, 2020) wie beispielsweise Gesundheitsämtern verwendet und kontinuierlich erweitert werden kann. Diese Lösung wäre zudem ein erster Schritt in Richtung eines deutschlandweiten virtuellen (Innovationsverbund Öffentliche Gesundheit 2021) oder digitalen Gesundheitsamtes (Bundesministerium für Gesundheit 2020).

Die erarbeiteten Artefakte und die verwendete Methodik sind dahingehend ein wichtiger Beitrag und können für die Skalierung des Vorgehens auf weitere Prozesse und Gesundheitsämter genutzt werden. Auf Grundlage der ausgearbeiteten Prozessmodule und Soll-Prozesse können im Weiteren Anforderungen an die zu entwickelnden Softwarekomponenten gestellt werden. Aufbauend auf den Prozessmodellen und den Anforderungen kann schließlich die Entwicklung der benötigten Schnittstellen, Softwarekomponenten und Infrastruktur erfolgen. Des Weiteren bieten die Soll-Prozesse einen Referenzpunkt bei der Erstellung prozessualer und technischer Richtlinien sowohl für bestehende als auch für neue Prozesse in den Gesundheitsämtern. Der Bereich der Wasserhygiene, in dem die Studie durchgeführt wurde, ist nur einer von mehreren Aufgabenbereichen der Gesundheitsämter. Um eine vollständige Transformation der bestehenden Prozesse durchzuführen, muss ein analoges Vorgehen in den restlichen Aufgabenbereichen von Gesundheitsämtern erfolgen. Die Studie zeigt exemplarisch auf, wie eine Identifizierung, Standardisierung, Modularisierung und Optimierung der Prozesse erfolgen kann und legt somit den zuständigen Experten des Gesundheitsamtes ein wichtiges Instrument für das weitere Vorgehen an die Hand.

Für die Schaffung eines deutschlandweiten virtuellen Gesundheitsamtes muss zudem über die Grenzen eines Gesundheitsamtes hinweg gedacht werden, da die Schaffung eines virtuellen Gesundheitsamtes eine weitestgehende Standardisierung und Modularisierung der bestehenden Prozesse in allen Gesundheitsämtern und allen Aufgabenbereichen erfordert. Eine detaillierte Prozesslandkarte des ÖGD in Deutschland in ihren Ist- und Soll-Zuständen würde ein solches Vorgehen unterstützen. Die Sammlung aller möglicher Prozessmodule würde einerseits einen Ausgangspunkt für die Entwicklung der Softwaremodule bieten und andererseits als Kompass bei der Umsetzung dieses Vorhabens dienen. Die Erarbeitung einer detaillierten Prozesslandkarte hat in Zusammenarbeit zwischen allen Gesundheitsämtern in Deutschland zu erfolgen. Hierbei müssen die Zuständigkeiten für diverse Aufgabenbereiche auf Gesundheitsämter verteilt werden. Die Erarbeitung der Soll-Prozesse sowie die Implementierung der Software für jedes Prozessmodul sollte sich dabei nach dem Einer-für-Alle (EfA) Prinzip richten (Bundesministerium des Innern und für Heimat 2021; Halsbenning 2021), wobei ein Modul von einem bestimmten Gesundheitsamt oder einer Gruppe von Gesundheitsämtern entwickelt und anschließend für alle bereitgestellt wird. Somit würde man durch kollaborative Zusammenarbeit eine Sammlung von passenden Softwaremodulen schaffen, an der sich alle Gesundheitsämter für die Zusammenstellung der benötigten Software bedienen könnten.

Die Ergebnisse dieser Studie dienen hierbei als Muster für die Vorgehensweise bei der Erarbeitung solcher Module. Im Kontext der Wasserhygiene können bereits jetzt aufbauend auf den Erkenntnissen des Gesundheitsamtes in Berlin die modularisierten Prozesse je nach Bedarf der Gesundheitsämter adaptiert werden. Die vorliegende Studie legt somit den Grundstein für die Vorgehensweise bei der Entwicklung des deutschlandweiten virtuellen Gesundheitsamtes und treibt die Digitalisierung des ÖGD voran. Darüber hinaus lässt sich das Vorgehen auf alle föderalen Prozesse im öffentlichen Sektor, die auf einer einheitlichen Gesetzesgrundlage basieren, übertragen. Dementsprechend können die Erkenntnisse aus dieser Studie genutzt werden, um die Digitalisierung der föderalen Strukturen und die Digitalisierung im Sinne des OZG und OZG 2.0 weiter voranzutreiben (Halsbenning 2021; Hogrebe 2021). Die Erkenntnisse auf Prozess- und Vorgehensebene werden derzeit über den Innovationsverbund Öffentliche Gesundheit mit Vertretern des ÖGD und der Politik auf Landes- und Bundesebene geteilt, um in der Folge eine zeitnahe und breite Umsetzung zu gewährleisten.

## Figures and Tables

**Abb. 3:**
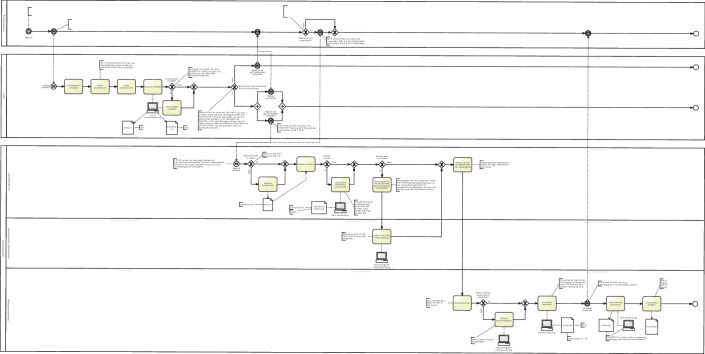
P8 – Untersuchung von Hausinstallationen auf Legionellen. Quelle: Eigene Darstellung

**Abb. 4:**
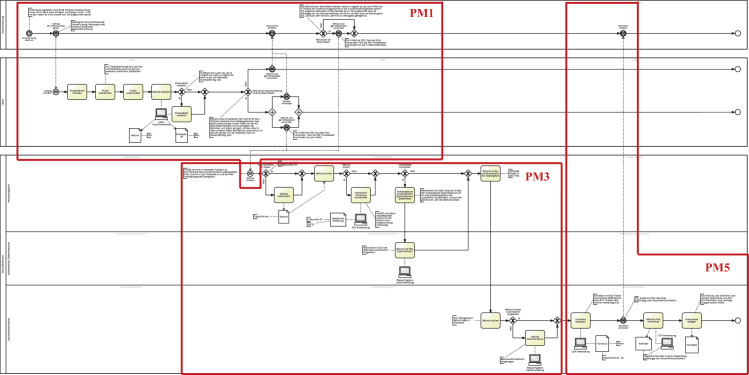
P8 – Modularisiert. Quelle: Eigene Darstellung

**Abb. 5:**
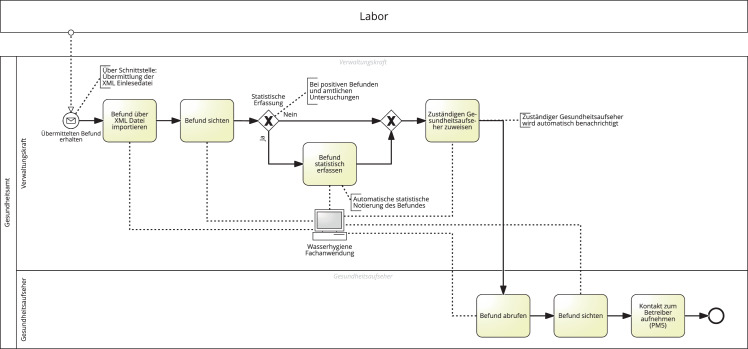
PM3 – Erfassung von einem Labor übermittelter Daten im Gesundheitsamt. Quelle: Eigene Darstellung
